# Sankofa pediatric HIV disclosure intervention did not worsen depression scores in children living with HIV and their caregivers in Ghana

**DOI:** 10.1186/s12889-020-09678-2

**Published:** 2020-10-20

**Authors:** Christopher Radcliffe, Aba Sam, Quinn Matos, Sampson Antwi, Kofi Amissah, Amina Alhassan, Irene Pokuaa Ofori, Yunshan Xu, Yanhong Deng, Nancy R. Reynolds, Elijah Paintsil, on Behalf of the Sankofa Team

**Affiliations:** 1 Department of Pediatrics, Pharmacology & Public Health, Yale School of Medicine, 464 Congress Avenue, New Haven, CT 06520 USA; 2 Department of Child Health, School of Medicine and Dentistry, Kwame Nkrumah University of Science and Technology and Komfo Anokye Teaching Hospital, Kumasi, Ghana; 3 Yale Center for Analytical Sciences, Yale School of Public Health, New Haven, CT USA; 4 Johns Hopkins School of Nursing, Baltimore, MD USA

**Keywords:** Pediatric HIV, Disclosure intervention, Depression, Ghana

## Abstract

**Background:**

The ‘Sankofa’ pediatric HIV disclosure study (2013–2017) was an intervention that aimed to address the low prevalence of disclosure of HIV status in Ghana.

**Methods:**

We conducted a cross-sectional study at the intervention site in Kumasi, Ghana, in 2019, (2 years after study closure) and administered the 21-item Beck Depression Inventory (BDI) and the 10-item Child Depression Inventory (CDI) to caregiver-child dyads who received the intervention.

**Results:**

We enrolled 65% (*N* = 157) of the original dyads in the present study. Between Sankofa enrollment baseline and the present study, both children and caregivers had significant (*p* < 0.0001) mean reductions in CDI scores and BDI scores, respectively. CDI scores of the children were significantly correlated with BDI scores of the caregivers (*r* = 0.019, *p* = 0.019). No statistically significant associations between disclosure status and either CDI score or BDI score were found.

**Conclusions:**

Our findings did not support caregivers’ fears that disclosure leads to depression.

**Trial registration:**

ClinicalTrials.gov Identifier: NCT01701635 (date of registration Oct 5, 2012).

## Background

In 2018, there were 2.8 million children and adolescents living with HIV globally [1]. Unfortunately, sub-Saharan Africa has been disproportionately affected by the HIV epidemic, and it is home to nearly 90% of children and adolescents living with HIV [1]. There has been an unprecedented effort to scale up antiretroviral therapy (ART) in sub-Saharan Africa. With expanding ART coverage in sub-Saharan Africa, children living with HIV are living longer and surviving into adulthood due to declines in AIDS-related deaths [2, 3]. However, these gains can be reversed when adherence to ART is sub-optimal. Sustained viral suppression is the goal of optimal adherence (≥95%), and there are several efforts and strategies focused on ensuring this level of adherence along the HIV care continuum, particularly in children living with HIV [4, 5]. Nevertheless, there are reports of poor adherence among children living with HIV leading to virologic or treatment failure [6, 7]. Non-disclosure of HIV status to children living with HIV is a critical barrier to optimal adherence to therapy [8]. There is a growing body of evidence to support the benefits of disclosure, such as retention in care, avoidance of treatment failure, and improvement of HIV knowledge [7, 9, 10].

Disclosure is the process by which a child is incrementally made aware of his or her HIV status in an age-appropriate manner [11]. Despite the WHO’s 2011 recommendations and guidelines that encourage disclosure, the prevalence of disclosure of HIV status to children living with HIV in sub-Saharan Africa is generally low, ranging from 9 to 72% in a recent review [11, 12]. A rate limiting factor for disclosure has been caregivers’ concerns that disclosure could have negative psychological and cognitive effects in children living with HIV [13, 14]. Other barriers center on caregivers’ fears that children living with HIV will tell others of their diagnoses, leading to stigmatization and isolation [13, 15, 16]. Moreover, there are no consensuses on who ought to disclose (caregiver vs. provider) and how to disclose. Few studies in sub-Saharan Africa have explored how best to disclose [14, 17, 18].

The ‘Sankofa’ pediatric HIV disclosure intervention was one such study that sought to address the low prevalence of disclosure in Ghana and the lack of disclosure skills among their caregivers [14, 15]. The site-randomized intervention lasted from January 2013 to June 2017, with a control site in Accra, Ghana, and an intervention site in Kumasi, Ghana. The intervention featured a disclosure model that incorporated bioecological systems theory and core elements of the Information-Motivation-Behavioral Skills model of Health Behavior Change [19–23]. The patient-centered intervention was delivered by adherence and disclosure specialists who were familiar with the community and trained to address the information, motivation, and behavioral skills of caregivers in an attempt to facilitate their engagement in the disclosure process from initiation to completion. The intervention was delivered in a sensitive fashion that considered the personal needs and cognitive capacities of children. Children in the intervention group were found to be 3.98- and 4.21-times more likely to have been disclosed to than children in the control group at one and 3 years, respectively [24].

We report the findings of a cross-sectional, post-intervention study conducted from June to August 2019 that sought to explore the long-term effects of the ‘Sankofa’ parent study and disclosure on depression symptoms in caregiver-child dyads at the intervention site, Kumasi, Ghana. We aimed to: 1) determine any changes in depression symptoms in dyads between Sankofa baseline and the current study and 2) explore the effect of disclosure status on depression symptoms over time.

## Methods

### Study participants

The recruitment and organization of the parent study, ‘Sankofa,’ has been previously described [14]. Study enrollment for the ‘Sankofa’ study lasted from January 2013 till June 2016, and the study ended in 2017. It had a control arm at Korle Bu Teaching Hospital in Accra, Ghana, and an intervention arm at Komfo Anokye Teaching Hospital (KATH) in Kumasi, Ghana. All caregiver-child dyads who received the intervention at KATH were eligible for inclusion in this cross-sectional study which enrolled participants from June to August 2019. Dyads were recruited at KATH’s pediatric HIV clinic during routine clinic appointments. Written informed consent was obtained from the caregivers of the children living with HIV, and consent was obtained from caregivers on behalf of children less than 18 years of age with an assent from the child participant. The study protocol was reviewed and approved by the Institutional Review Boards of Kwame Nkrumah University of Science Technology/KATH (Protocol #: CHRPE/AP/146/12) and Yale University (Protocol #: 1205010310).

### Procedures

The 10-item Child Depression Inventory (CDI) and the 21-item Beck Depression Inventory (BDI) were administered to caregiver-child dyads in the parent Sankofa study every 48 weeks till the end of the study [25]. Other instruments such as Brief Illness Perceptions Questionnaire (Brief IPQ), Social Provisions Scale, brief HIV Knowledge questionnaire (HIV-KQ-18), and HIV Stigma Scale were administered to caregivers during the same timepoints in the parent study [26–29]. In the current study, we administered only the CDI and BDI to the children and caregivers, respectively. To avoid accidental disclosure, questionnaires were administered to children in the presence of their caregivers whereas caregivers were interviewed alone. The interviews took place in a clinic setting, and the majority were completed in English. For participants who only spoke Twi, the major regional language, native-speaking research staff translated accordingly. The original research staff from the Sankofa study administered the questionnaires with the help of authors AS, QM, and CR.

### Measures

Demographic data for the caregiver-child dyads were collected upon enrollment in the Sankofa study. The 10-item CDI was used to screen for depression symptoms in children. Each of the 10 items is scored on a four-point scale (0–3) to yield a maximum score of 30. This abridged form of the CDI has yet to be validated in our population; however, a version of the 10-item CDI has been studied in African adolescents without HIV [30]. Cutoff scores are not available, and a higher CDI score indicates greater severity of depression symptoms. The 21-item BDI was used to assess depression symptoms in caregivers. The BDI has been previously validated in sub-Saharan Africa [31], and it rates each of its 21 items on a four-point scale (0–3). A total score is assigned, and its results are divided into four categories depending on the severity of depression symptoms: minimal (0–9); mild (10–18); moderate (19–29); and severe (30–63). For our population, the BDI has been reported to have a three-factor structure (affective, cognitive, and somatic) and high internal reliability (0.90) [31].

### Analysis

Baseline demographic data were summarized with descriptive statistics. Means and standard deviations were determined for continuous variables whereas counts and percentages were determined for categorical variables. Differences in demographic data between disclosed and non-disclosed groups were assessed using t-test or Wilcoxon rank sum test for continuous variables and chi-square or Fisher’s exact tests for categorical variables, as appropriate. The same methods were used to assess differences between dyads who received the intervention and were enrolled in the current study versus those who were not enrolled. The mean changes of CDI and BDI scores between baseline and the current study were examined using paired t-tests. The correlation of CDI and BDI scores was examined using Pearson’s correlation coefficients. The changes in BDI categories, defined by standard cutoff scores, were also evaluated using McNemar’s test. Similar analysis for CDI data was not included due to lack of validated cutoff scores for categorizing symptom severity. Effect of disclosure status on CDI and BDI scores over major assessment time points was evaluated separately using repeated measures analysis with disclosure status entered as time varying covariate [32]. Baseline CDI/BDI score, time, and interaction between time and disclosure status were also included as fixed effects for both models. Two more covariates, baseline HIV Stigma Scale score and Brief IPQ score, were included in the model for BDI score. *P* value less than 0.05 was considered statistically significant. All analyses were conducted using SAS/STAT® software, Version 9.4 of the SAS System for Windows (Cary, NC, USA).

## Results

### Characteristics of study participants

Of the 240 caregiver-child dyads who received the Sankofa intervention at KATH during the parent study, 65% (*N* = 157) of them were successfully enrolled in the current study. Of the remaining 83 caregiver-child dyads, 51 dyads were nonresponses—they did not attend clinic during the study period and/or did not respond to phone calls to invite participation. The remaining dyads (*N* = 32) were excluded for the following reasons: child passed (*N* = 18); caregiver passed (*N* = 11); both child and caregiver passed (*N* = 3). Table 1 summarizes the children’s demographics; 50.3% were female, and the median age at enrollment was 10 years (IQR 8–12). The majority attended school (93%), and mother to child transmission was the most common mode of HIV transmission (79.6%). The disclosed group was significantly older (*p* < 0.001), had been on ART for a longer period of time (*p* = 0.014), and had a longer mean time between enrollment and the current study (*p* < 0.001). No significant differences were noted between children who were or were not enrolled in this study.

**Table 1:** Child demographics

	Disclosure Status			
	No (***N*** = 35)	Yes (***N*** = 122)	Total (***N*** = 157)	***p*** Value
**Gender**				
Female	019 (54.29%)	060 (49.18%)	079 (50.32%)	
Male	016 (45.71%)	062 (50.82%)	078 (49.68%)	0.59 ^a^
**Child Age**				
Mean (95% CL)	8.88 (8.25–9.51)	10.31 (9.86–10.75)	9.99 (9.61–10.37)	< 0.001^c,^ ***
Median (IQR)	9 (7–10)	10 (8–12)	10 (8–12)	
N (N Missing)	33 (2)	118 (4)	151 (6)	
**School**				
Yes	030 (85.71%)	116 (95.08%)	146 (92.99%)	
No	002 (5.71%)	001 (0.82%)	003 (1.91%)	
Patient refused to answer	000 (0.00%)	001 (0.82%)	001 (0.64%)	
Missing	003 (8.57%)	004 (3.28%)	007 (4.46%)	0.20 ^b^
**HIV Transmission Mode**				
MTC	025 (71.43%)	100 (81.97%)	125 (79.62%)	
Other	001 (2.86%)	003 (2.46%)	004 (2.55%)	
Missing	009 (25.71%)	019 (15.57%)	028 (17.83%)	1.00 ^b^
**Duration of HIV (days)**				
Mean (95% CL)	1503.60 (1006.07–2001.13)	1749.07 (1545.42–1952.71)	1702.58 (1514.83–1890.32)	0.31 ^c^
Median (IQR)	1264 (406–2513)	1928 (747–2588)	1898 (745–2579)	
N (N Missing)	25 (10)	107 (15)	132 (25)	
**Duration of ART treatment (days)**				
Mean (95% CL)	804.92 (381.21–1228.64)	1355.88 (1162.56–1549.19)	1246.53 (1068.69–1424.37)	0.014 ^c,^ *
Median (IQR)	350 (32–1208)	1389 (406–2145)	1281 (147–2107)	
N (N Missing)	26 (9)	105 (17)	131 (26)	
**WHO Staging at time of Diagnosis**				
Stage 1	003 (8.57%)	016 (13.11%)	019 (12.10%)	
Stage 2	006 (17.14%)	044 (36.07%)	050 (31.85%)	
Stage 3	014 (40.00%)	030 (24.59%)	044 (28.03%)	
Stage 4	002 (5.71%)	013 (10.66%)	015 (9.55%)	
Missing	010 (28.57%)	019 (15.57%)	029 (18.47%)	0.10 ^a^
**HIV+ caregiver**				
Yes	017 (48.57%)	079 (64.75%)	096 (61.15%)	
No or Unsure	017 (48.57%)	040 (32.79%)	057 (36.31%)	
Missing	001 (2.86%)	003 (2.46%)	004 (2.55%)	0.08 ^a^
**Does anyone help you take your medicine?**				
Yes	024 (68.57%)	101 (82.79%)	125 (79.62%)	
No	006 (17.14%)	012 (9.84%)	018 (11.46%)	
Missing	005 (14.29%)	009 (7.38%)	014 (8.92%)	0.21 ^a^
**Who helps you with your medicine most of the time?**				
Biological parent (Mother/Father)	017 (48.57%)	075 (61.48%)	092 (58.60%)	
Family (Grandparent/Aunt/Uncle)	006 (17.14%)	013 (10.66%)	019 (12.10%)	
Other family member	001 (2.86%)	010 (8.20%)	011 (7.01%)	
Other	000 (0.00%)	002 (1.64%)	002 (1.27%)	
Unknown	000 (0.00%)	001 (0.82%)	001 (0.64%)	
Missing	011 (31.43%)	021 (17.21%)	032 (20.38%)	0.55 ^b^
**Days since disclosure**				
Mean (95% CL)	NA (NA – NA)	1540.62 (1467.34–1613.90)	1540.62 (1467.34–1613.90)	
Median (IQR)	NA (NA – NA)	1573 (1215–1834)	1573 (1215–1834)	
N (N Missing)	0 (35)	121 (1)	121 (36)	
**Days since enrollment**				
Mean (95% CL)	1603.26 (1461.08–1745.43)	1914.43 (1851.38–1977.48)	1845.06 (1783.96–1906.15)	< 0.001^c,^ ***
Median (IQR)	1519 (1238–1921)	1941 (1624–2243)	1893 (1519–2215)	

Legend: * *p* < 0.05, *** *p* < 0.001

^a^ Chi-square test, ^b^ Fisher’s Exact test, ^c^ T test

Abbreviations: *ART* antiretroviral therapy; *CL* confidence level; *IQR* interquartile range; *MTC* mother-to-child

The demographics of the caregivers are presented in Table 2. Caregivers’ mean age was 42.22 ± 9.69 years, and 82.8% were female. Fifty-six percent of caregivers were married, and most were living with HIV (61.2%). Caregivers in the disclosed group were significantly older (*p* = 0.04) and differed in their employment statuses (*p* = 0.020). Compared to caregivers who were enrolled in the current study, those who were not had significant differences in marital status (*p* = 0.016), monthly household income (*p* = 0.036), and baseline HIV-KQ-18 score (*p* = 0.018). Compared to the caregivers not enrolled in the current study, the caregivers enrolled lived significantly closer to KATH (*p* = 0.046).

**Table 2:** Caregiver demographics

	Disclosure Status of Child			
	No (***N*** = 35)	Yes (***N*** = 122)	Total (***N*** = 157)	***p*** Value
**Age caregiver**				
Mean (SD)	39.29 (10.22)	43.07 (9.41)	42.22 (9.69)	0.042 ^c,^ *
**Marital Status**				
Divorced or separated	002 (5.71%)	011 (9.02%)	013 (8.28%)	
Married	019 (54.29%)	069 (56.56%)	088 (56.05%)	
Single	007 (20.00%)	014 (11.48%)	021 (13.38%)	
Widowed	007 (20.00%)	028 (22.95%)	035 (22.29%)	0.58 ^a^
**Gender**				
Female	28 (80.00%)	102 (83.61%)	130 (82.80%)	
Male	7 (20.00%)	20 (16.39%)	27 (17.20%)	0.59 ^a^
**Caregiver HIV status**				
No or Unsure	017 (48.57%)	040 (32.79%)	057 (36.31%)	
Yes	017 (48.57%)	079 (64.75%)	096 (61.15%)	
Missing	001 (2.86%)	003 (2.46%)	004 (2.55%)	0.08 ^a^
**Education**				
No School	006 (17.14%)	016 (13.11%)	022 (14.01%)	
Elementary Education	023 (65.71%)	086 (70.49%)	109 (69.43%)	
Secondary & Post-secondary Education	006 (17.14%)	017 (13.93%)	023 (14.65%)	
Missing	000 (0.00%)	003 (2.46%)	003 (1.91%)	0.75 ^a^
**Monthly Household Income**				
< 50 GHS	04 (11.43%)	19 (15.57%)	23 (14.65%)	
50–300 GHS	23 (65.71%)	78 (63.93%)	101 (64.33%)	
> 300 GHS	8 (22.86%)	23 (18.85%)	31 (19.75%)	
Missing	0 (0.00%)	2 (1.64%)	2 (1.27%)	0.76 ^a^
**Employment Status**				
Unemployed	10 (28.57%)	13 (10.66%)	23 (14.65%)	
Self-employed	4 (11.43%)	10 (8.20%)	14 (8.92%)	
Private/Government Sector	21 (60.00%)	99 (81.15%)	120 (76.43%)	0.020 ^a,^ *
**Family members/other people in home (Child)**				
Mean (SD)	4.06 (2.24)	3.84 (3.36)	3.89 (3.13)	0.66 ^c^
N (N Missing)	35 (0)	121 (1)	156 (1)	
**Family members/other people in home (Adult)**				
Mean (SD)	3.63 (2.29)	4.46 (3.70)	4.27 (3.44)	0.11 ^c^
N (N Missing)	35 (0)	118 (4)	153 (4)	
**Number of living children**				
Mean (SD)	3.19 (1.87)	2.90 (1.88)	2.96 (1.87)	0.44 ^c^
N (N Missing)	32 (3)	120 (2)	152 (5)	
**Distance from Clinic/Hospital**				
0–10 km	013 (37.14%)	046 (37.70%)	059 (37.58%)	
10–20 km	015 (42.86%)	044 (36.07%)	059 (37.58%)	
20–30 km	004 (11.43%)	016 (13.11%)	020 (12.74%)	
More than 30 km	003 (8.57%)	014 (11.48%)	017 (10.83%)	
Missing	000 (0.00%)	002 (1.64%)	002 (1.27%)	0.90 ^a^
**Receiving own care at**				
Clinic/Hospital and traditional/homeopathic healer	000 (0.00%)	001 (0.82%)	001 (0.64%)	
Clinic/Hospital	035 (100.00%)	120 (098.36%)	155 (98.73%)	
Missing	000 (0.00%)	001 (0.82%)	001 (0.64%)	0.59 ^b^
**Frequency of visits to clinic/hospital**				
Once or never in last 5 years	011 (31.43%)	045 (36.89%)	056 (35.67%)	
One or more times a year	024 (68.57%)	075 (61.48%)	099 (63.06%)	
Other	000 (0.00%)	001 (0.82%)	001 (0.64%)	
Missing	000 (0.00%)	001 (0.82%)	001 (0.64%)	0.70 ^b^
**Social Provisions Questionnaire Overall Score**				
Mean (SD)	71.86 (5.75)	71.36 (5.95)	71.47 (5.89)	0.66 ^c^
N (N Missing)	35 (0)	121 (1)	156 (1)	
**HIV Knowledge (HIV-KQ-18)**				
Mean (SD)	14.54 (1.93)	14.70 (1.90)	14.67 (1.90)	0.66 ^c^
N (N Missing)	35 (0)	118 (4)	153 (4)	
**HIV Stigma Score**				
Mean (SD)	39.89 (6.48)	41.24 (4.88)	40.94 (5.29)	0.26 ^c^
N (N Missing)	35 (0)	120 (2)	155 (2)	
**Illness Perception Score (Brief IPQ)**				
Mean (SD)	31.34 (11.25)	32.25 (11.51)	32.05 (11.42)	0.68 ^c^
N (N Missing)	35 (0)	120 (2)	155 (2)	

Legend: 1.0 Ghanaian cedi is equivalent to 0.17 U.S. dollar; * *p* < 0.05

^a^ Chi-square test, ^b^ Fisher’s Exact test, ^c^ T test

Abbreviations: *GHS* Ghanaian cedi; *SD* standard deviation

### Depression scores at ‘Sankofa’ baseline and the current study in children living with HIV and their caregivers

In the current study, participants’ depression scores were collected a median of 5.19 years (IQR 4.16–6.07 years) after participants’ respective dates of enrollment in the parent study. The CDI scores of the children were 5.19 ± 3.77 (mean ± SD) and 3.35 ± 3.50 (mean ± SD) at ‘Sankofa’ baseline and the current study, respectively. There was a significant mean reduction of 1.82 (95% CI: 1.01–2.63) between baseline and the current study’s measurements (*p* < 0.0001). Interestingly, children who were disclosed to had a greater reduction in CDI scores compared to those who were not disclosed to, although, this did not reach statistical significance. After adjusting for time since enrollment, we still found no significant difference in CDI reduction between disclosed and non-disclosed children (*p* = 0.19). CDI scores of the children in the current study were significantly correlated with BDI scores of the caregivers in the current study (*r* = 0.19, *p* = 0.019).

BDI scores at ‘Sankofa’ baseline and the current study for caregivers were 6.55 ± 6.02 and 3.94 ± 4.49, respectively. We observed a statistically significant reduction in BDI scores between baseline and the current study, (2.65 (95% CI: 1.76–3.55), *p* < 0.0001). However, we did not observe any significant difference in mean reduction between caregivers of disclosed children compared to caregivers of non-disclosed children. When adjusting for time since enrollment for caregivers, we still did not find a significant difference in BDI reduction between caregivers of disclosed and non-disclosed children (*p* = 0.41). BDI scores from baseline and the current study were found to be highly correlated (*r* = 0.45, *p* < 0.0001). When BDI scores were analyzed as a categorical variable, we observed improvement of scores between baseline and the current study: 6 (86%) caregivers with mild depression changed to minimal depression; 5 (100%) caregivers with moderate depression changed to mild or minimal depression, and 2 (100%) caregivers with severe depression changed to moderate or minimal depression over time. Only 2 (1.5%) caregivers changed to mild or moderate depression from minimal depression. These changes did not reach statistical significance (Table 3). However, when the categories were combined into minimal versus mild, moderate, and severe depression, the changes reached statistical significance (*p* = 0.02). We repeated this analysis by disclosure status and found borderline significant improvement in the caregivers of disclosed children (*p* = 0.06) but not in the caregivers of undisclosed children (*p* = 0.16).

**Table 3:** Changes in Beck Depression Inventory categories between baseline and the current study

Baseline BDI Categories	Current Study BDI Categories					***p*** value
	Minimal (No.)	Mild (No.)	Moderate (No.)	Severe (No.)	Total (No.)	
**Minimal (No.)**	140	1	1	0	142	0.10 ^a^
**Mild (No.)**	6	0	1	0	7	
**Moderate (No.)**	3	2	0	0	5	
**Severe (No.)**	1	0	1	0	2	
**Total (No.)**	150	3	3	0	156	

Legend: ^a^ Generalized McNemar’s test; Abbreviation: *BDI* Beck Depression Inventory

### Longitudinal trends in depression scores in children living with HIV and their caregivers

**Fig. 1:**
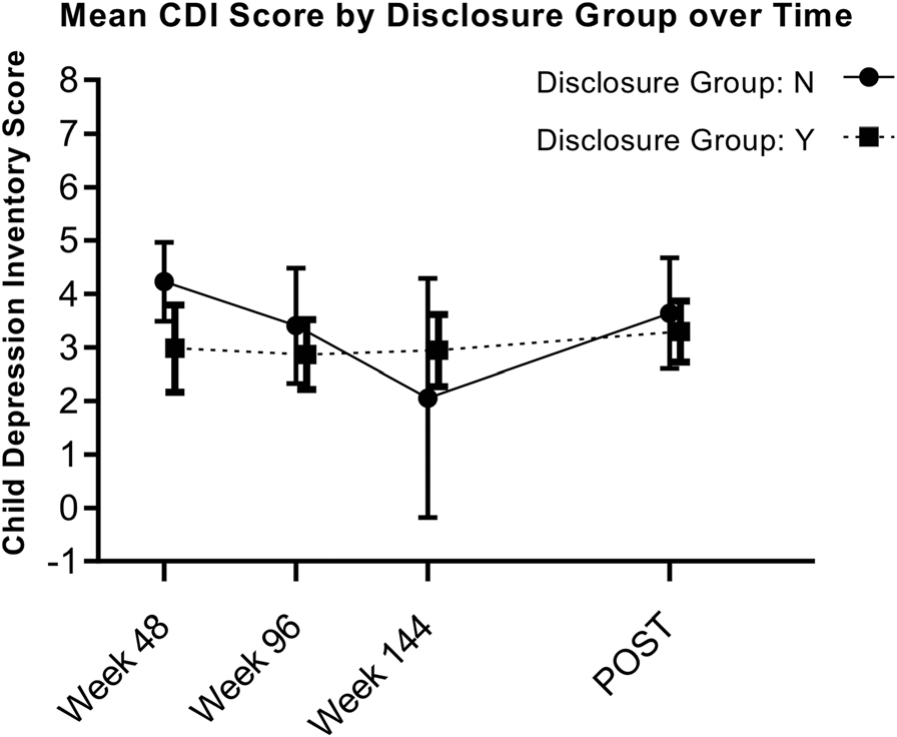
Depression symptoms in children by disclosure group over time. Disclosure groups represent children who did or did not complete the disclosure process at each time point. Child Depression Inventory (CDI) scores for weeks 48, 96, and 144 were collected during the Sankofa parent study. *POST* data point was collected during the current study and represents time between enrollment in the parent study and the current study, which varies for study participants (median 5.19 years; IQR 4.16–6.07 years). Effect of disclosure status on CDI scores was evaluated using repeated measures analysis with disclosure status entered as time varying covariate. Abbreviation: CDI, Child Depression Inventory

Overall, we did not find a statistically significant association between disclosure status and CDI score of children (*p* = 0.48). The adjusted mean CDI score of children who were disclosed to was lower than that of children who were not disclosed to across all major assessment time points except week 144. Only at week 48 was the mean difference statistically significant (Fig. 1, Table 4).

**Table 4:** Least square means for depression scores over time

Week	Disclosed LSM (95% CI)	Non-disclosed LSM (95% CI)	Difference of LSM (95% CI)	***p*** value
**Least Square Means of BDI**				
48	5.31 (4.43, 6.19)	3.69 (2.69, 4.68)	1.62 (0.39, 2.85)	0.01 ^a,^ *
96	4.8 (3.52, 6.08)	4.21 (3.38, 5.04)	0.59 (−0.86, 2.04)	0.42 ^a^
144	4.17 (1.74, 6.59)	3.76 (2.91, 4.62)	0.4 (−2.12, 2.93)	0.75 ^a^
post	4.36 (3.09, 5.63)	3.64 (2.9, 4.38)	0.72 (−0.69, 2.13)	0.32 ^a^
**Least Square Means of CDI**				
48	4.23 (3.49, 4.97)	2.98 (2.17, 3.8)	1.25 (0.2, 2.29)	0.02 ^a,^ *
96	3.4 (2.33, 4.48)	2.87 (2.22, 3.52)	0.54 (−0.68, 1.76)	0.39 ^a^
144	2.06 (−0.18, 4.29)	2.95 (2.27, 3.62)	−0.89 (−3.2, 1.43)	0.45 ^a^
post	3.64 (2.61, 4.68)	3.3 (2.73, 3.87)	0.34 (−0.81, 1.5)	0.56 ^a^

Legend: * *p* < 0.05, ^a^ T test; Abbreviations: *BDI* Beck Depression Inventory; *CDI* Child Depression Inventory; *CI* confidence interval; *LSM* least square means

**Fig. 2:**
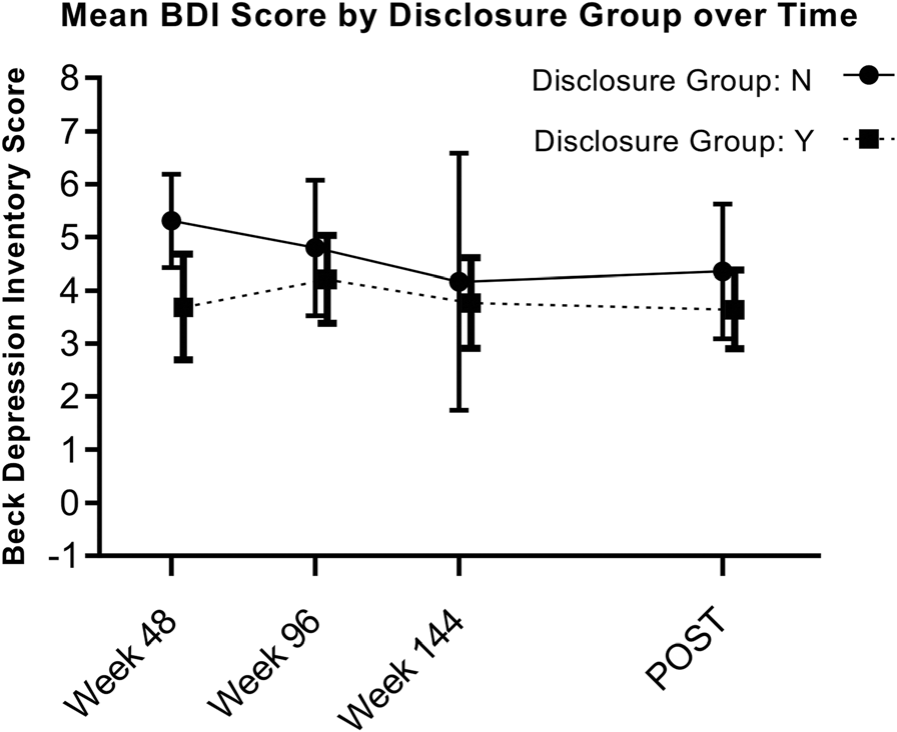
Depression symptoms in caregivers by disclosure group over time. Disclosure groups represent caregivers of children who did or did not complete the disclosure process at each time point. Beck Depression Inventory (BDI) scores for weeks 48, 96, and 144 were collected during the Sankofa parent study. *POST* data point was collected during the current study and represents time between enrollment in the parent study and the current study, which varies for study participants (median 5.19 years; IQR 4.16–6.07 years). Effect of disclosure status on BDI scores was evaluated using repeated measures analysis with disclosure status entered as time varying covariate. Abbreviation: BDI, Beck Depression Inventory

A separate mixed model was created for BDI scores of caregivers with baseline HIV Stigma Scale score and Brief IPQ score added as covariates. We did not find a statistically significant association between disclosure status of children and BDI score (*p* = 0.11). Compared to caregivers of non-disclosed children, the adjusted mean BDI score of caregivers of disclosed children was lower at all time points during the parent study and the current study; however, this was not statistically significant except at week 48 (Fig. 2, Table 4).

## Discussion

We conducted a cross-sectional study on caregiver-child dyads a median of 5.19 years (IQR 4.16–6.07 years) after their enrollment in the parent ‘Sankofa’ study at the intervention site in Kumasi, Ghana, which ended in June 30, 2017. Using screening instruments for depression symptoms, we found statistically significant (*p* < 0.0001) reductions in mean scores for both children living with HIV and their caregivers when compared to baseline values. Contrary to commonly held beliefs in resource-limited settings, there was no increase in depression symptoms amongst caregiver-child dyads who received a clinic-based, culturally sensitive disclosure intervention. Our findings suggest that intervention-assisted disclosure can allay caregivers’ fears surrounding the potential for negative psychological effects after disclosure of HIV status to children living with HIV.

We found a significant mean reduction of 1.82 (95% CI: 1.01–2.63; *p* < 0.0001) in CDI scores between baseline and the current study’s measurements, a median of 5.19 years (IQR 4.16–6.07 years) after enrollment. This finding suggests that a culturally and developmentally sensitive disclosure process did not worsen depression symptoms. In contrast, a recent report on a disclosure intervention in Kenya showed that children in the intervention arm had higher odds of moving from a less to a more severe depression category on the Patient Health Questionnaire-9 at a time when the prevalence of disclosure in the study cohort had increased [33, 34]. Notably, the difference in odds between the control and intervention arms nearly resolved by the study’s last measurement [34], suggesting that the risk of depression is present in the months following disclosure then resolves over time. We did not observe this trend in our data. This likely stems from the proactive nature of the ‘Sankofa’ study. The trained adherence and disclosure specialists who initiated the disclosure process continued counseling children after disclosure, and the study offered 3 years of intensive psychosocial support [14]. In settings with limited access to mental health resources, an emphasis on primary prevention is needed, and the Sankofa study reflects this. Only our week 48 (12 months) measurement showed a statistically significant difference between groups, with the disclosed group having a lower mean CDI score. Depression symptoms in children at our intervention site actually improved between enrollment and the current study.

The challenges of caring for children living with HIV and the impact of caregivers’ mental health on children make assessment of caregivers’ depression symptoms equally important [35, 36]. In the present study, we observed statistically significant mean reductions (*p* < 0.0001) in BDI scores between baseline and the current study’s measurements. At each time point, adjusted mean BDI scores were consistently lower for caregivers of children who had completed the disclosure process. Caregivers of children living with HIV have been found to have a high prevalence of depression symptoms, and our findings suggest disclosure may help to alleviate the burden in this high-risk group [37, 38]. Interestingly, we also found CDI scores in the current study to be significantly correlated with the BDI scores of caregivers (*r* = 0.19, *p* = 0.019). This finding is encouraging because the ‘Sankofa’ intervention intentionally focused on the experience of both children and their caregivers, with the disclosure process emphasizing inclusion and mutual empowerment. Caregiver depression symptoms being positively associated with those of children has been previously reported [39], and our observations demonstrate that methods can be successfully developed and deployed to benefit both caregivers and children.

When interpreting our results, there are limitations one needs to consider. First, this exploratory study was only conducted at the intervention site in Kumasi, Ghana. Due to logistical and temporal constraints, we could not parallel the original design of the ‘Sankofa’ study; however, the non-disclosed children in the current study serve as controls for the disclosed children because all dyads received the intervention. Our findings show sustained reductions in depression scores for caregiver-child dyads years after the intervention. A second limitation is the use of screening instruments to assess depression symptoms in caregiver-child dyads. They are time-efficient and provide valuable information when analyzed longitudinally, but they are no substitute for a thorough psychiatric evaluation and may be subject to reporting bias. Finally, a number of dyads (*N* = 83) who received the intervention were not included in this cross-sectional study for various reasons. Compared to caregivers who were not enrolled in this current study, enrolled caregivers lived closer to KATH (*p* = 0.046). Most of the enrolled caregivers lived within urban Kumasi, and the heterogeneity of the surrounding areas may not be adequately captured by our study. This study took place over a finite period of time, and limitations on the ability to enroll all original participants does not undermine our findings. Notably, there were no significant differences between the children, and this strengthens our confidence in the results.

## Conclusions

As the prognosis for children living with HIV changes with the expansion of ART, strategies for improving adherence and engagement with care must be developed. Age-appropriate disclosure of HIV status has been recommended by the American Academy of Pediatrics and the WHO [40, 11], and our study supports this recommendation’s applicability in sub-Saharan Africa. This region is regrettably burdened with 90% of all children and adolescents living with HIV [1], but it will benefit the most from disclosure interventions which engage children and their caregivers.

## Data Availability

The datasets used and analyzed during the current study are available from the corresponding author on reasonable request.

## Abbreviations

ART: Antiretroviral therapy
BDI: Beck Depression Inventory
CDI: Child Depression Inventory
IPQ: Illness Perceptions Questionnaire
KATH: Komfo Anokye Teaching Hospital
WHO: World Health Organization
